# Reconstruction and Analysis of the Immune-Related LINC00987/A2M Axis in Lung Adenocarcinoma

**DOI:** 10.3389/fmolb.2021.644557

**Published:** 2021-04-27

**Authors:** Jiakang Ma, Xiaoyan Lin, Xueting Wang, Qingqing Min, Tonglian Wang, Chaozhi Tang

**Affiliations:** ^1^Department of Oncology, The Second Affiliated Hospital of Zhengzhou University, Zhengzhou, China; ^2^Department of Ophthalmology, The First Affiliated Hospital and College of Clinical Medicine of Henan University of Science and Technology, Luoyang, China; ^3^Department of Endodontics, Stomatological Hospital of China Medical University, Shenyang, China; ^4^Research Center of Molecular Medicine of Yunnan Province, Faculty of Life Science and Technology, Kunming University of Science and Technology, Kunming, China; ^5^Department of Urology, The First Affiliated Hospital of China Medical University, Shenyang, China

**Keywords:** eRNA, LUAD, LINC00987/A2M axis, immune cell infiltration, tumor hypoxia, tumor cell stemness

## Abstract

Enhancer RNAs (eRNAs) participate in tumor growth and immune regulation through complex signaling pathways. However, the immune-related function of the eRNA-mRNA axis in lung adenocarcinoma (LUAD) is unclear. Data on the expression of eRNAs and mRNAs were downloaded from The Cancer Genome Atlas, GEO, and UCSC Xena, including LUAD, and pan-cancer clinical data and mutational information. Immune gene files were obtained from ImmLnc and ImmPort databases. Survival indices, including relapse-free and overall survival, were analyzed using the Kaplan–Meier and log-rank methods. The level of immune cell infiltration, degree of tumor hypoxia, and tumor cell stemness characteristics were quantified using the single-sample gene set enrichment analysis algorithm. The immune infiltration score and infiltration degree were evaluated using the ESTIMATE and CIBERSORT algorithms. The tumor mutation burden and microsatellite instability were examined using the Spearman test. The LUAD-associated immune-related LINC00987/A2M axis was down-regulated in most cancer types, indicating poor survival and cancer progression. Immune cell infiltration was closely related to abnormal expression of the LINC00987/A2M axis, linking its expression to a possible evaluation of sensitivity to checkpoint inhibitors and response to chemotherapy. Abnormal expression of the LINC00987/A2M axis was characterized by heterogeneity in the degree of tumor hypoxia and stemness characteristics. The abnormal distribution of immune cells in LUAD was also verified through pan-cancer analysis. Comprehensive bioinformatic analysis showed that the LINC00987/A2M axis is a functional and effective tumor suppressor and biomarker for assessing the immune microenvironment and prognostic and therapeutic evaluations of LUAD.

## Introduction

Non-small-cell lung carcinoma (NSCLC) is the main histological type accounting for approximately 85% of lung cancer (LC) patients (van Meerbeeck et al., 2011; Domingues et al., 2014). Of the 70% of NSCLC patients diagnosed with advanced or metastatic progression, only 30% of cases are operable (Travis et al., 2013). The most common type of NSCLC is lung adenocarcinoma (LUAD), which reportedly comprises 40% of all LC and 60% of NSCLC (Meza et al., 2015; Torre et al., 2016).

Emerging evidence has emphasized the critical enhancer roles, a key non-coding DNA sequence in promoter-distal *cis*-regulatory DNA regions, in cancer genomic studies (Pennacchio et al., 2013). Enhancer RNAs (eRNAs) are a type of bidirectional RNA transcript originating during enhancer activation and transcription. In the process, enhancers attract, and bind transcription factors (TFs) by exposing DNA motifs of local chromatin, and TFs further enlist RNA Pol II to induce eRNA transcription (Kim et al., 2010; Heinz et al., 2015; Murakawa et al., 2016). eRNA is categorized as a type of long non-coding RNA (lncRNA) based on its length (Li et al., 2016). As many eRNAs have shown to be too unstable and low in abundance, they are rarely observed via steady-state RNA assays such as RNA-seq and thus cannot be completely captured in lncRNA databases (Derrien et al., 2012). Therefore, it is of great research significance to find an eRNA that exhibits overlap with lncRNAs. In particular, transcription of eRNA is known to be initiated from TF binding sites and has been reported to act as a robust readout predicting the activity of TFs (Azofeifa et al., 2018). Some studies have offered strong evidence that eRNAs could be induced to participate in regulating the activation of oncogenes or oncogenic signaling pathways, such as ESR1 (Li et al., 2015), and that of repressors such as TP53 (Melo et al., 2013) and Rev-Erbs (Lam et al., 2013). Besides, eRNA has shown to be functional in stimulating transcription of target mRNAs (Kim et al., 2010; Zhang et al., 2019). eRNAs have been found to promote transcription by establishing chromatin remodeling and Pol II assembly at defined loci, thereby suggesting higher expression of genes near eRNA + sites as compared to that at other sites (Mousavi et al., 2013). However, the functions and mechanisms of eRNAs with target mRNAs remain incompletely understood in LUAD.

Effective immunotherapies that induce or enhance antitumor responses have prospective clinical applications in the treatment of cancer. For example, combination therapy with immune checkpoint inhibitors of cytotoxic T lymphocyte-associated protein 4 (CTLA) and programmed cell death 1 (PDCD1, also known as PD1) has shown effective clinical application potential in the treatment of cancer (Liu J.-N. et al., 2020). Although the study of the molecular identification of tumor antigens for therapeutic anticancer vaccines is more urgent, the underlying active immunoregulatory processes in the tumor microenvironment are not negligible. Further, eRNAs correlated with six immune checkpoint proteins (CD200, PLEC, PDL2, HAVCR1, PDL1, and BTLA) in at least five cancer types (Zhang et al., 2019). They were suggested to play vital roles in evading immune destruction and maintaining the balance between activation of adaptive immunity and self-tolerance of autoimmunopathy. The associated transcript 1-long isoform (CCAT1-L) is a super-enhancer RNA known to positively regulate the expression of MYC in *cis*, which is a key regulator of the immune checkpoint (CD47 and PDCD1L1), immune surveillance, and antitumor immune response (Casey et al., 2016; Kim et al., 2017; Wu and Shen, 2019). In addition, eRNAs have been closely related to the development and release of inflammatory mediators in immune cells. For instance, IL1b-eRNA was found to decrease the lipopolysaccharide (LPS)-induced release of interleukin 1 beta (IL1b) and C-X-C motif chemokine ligand 8 (CXCL8) in human monocytes, which are suggested to be important regulators of the human innate immune response (Iiott et al., 2014). Likewise, LNCGme02323 was demonstrated to drastically alter the expression of marginal zone B cells. Briefly, LNCGme00432, LNCGme00344, and LNCGme00345 are known to be regulated by PAX5- and PAX5-dependent pathways, which might contribute to the differentiation of acute B lymphoblastic leukemia (B-ALL) cells and leukemia regression (Brazão et al., 2016). Therefore, continued detailed analysis of immune cell infiltration in the tumor microenvironment could help identify potential biomarkers and develop new immunotherapeutic strategies.

The therapeutic trait of atezolizumab, the PDL-1 blocking antibody used for the treatment of NSCLC, is currently under investigation (Ryu and Ward, 2018) and is thus still unavailable for clinical application. We aimed to explore more functional immunomodulators that can provide a powerful basis for immunotherapy of LUAD in the future. This study identified the immune-related eRNA-mRNA axis using data obtained from The Cancer Genome Atlas (TCGA), ImmLnc, and ImmPort databases and found that this axis plays a vital role in clinical cancer progression. We further explored the relationship between the eRNA-mRNA axis and the level of immune cell infiltration, degree of tumor hypoxia, tumor cell stemness, somatic mutations, and prognostic values for patients in LUAD TCGA and 3 GEO datasets. To verify the significance of the eRNA-mRNA axis in immunotherapy, a pan-cancer evaluation was conducted for tumor mutation burden (TMB), microsatellite instability (MSI), and immune cell populations. These results provided strong evidence for the role of eRNAs in regulating immune status and immunotherapy.

## Materials and Methods

### Data Collection and Processing

Sequencing data (FPKM) and clinical data of LUAD included the datasets of 491 cases obtained from the TCGA data portal^1^. Pan-cancer and somatic mutation data in LUAD were downloaded from the UCSC Xena^2^ (Table 1). Three GEO datasets, GSE31210, GSE37745, and GSE50081, containing the microarray-based expression data of patients with LUAD and associated clinical information, were downloaded from the GEO website^3^. GSE31230 (*N* = 226) was annotated using the data file for Agilent-021169 Arabidopsis 4 Oligo Microarray (V4; Feature Number version). The GSE37745 and GSE50081 datasets were filtered, and only LUAD cases were retained, following which both were annotated using the data files from the [HG-U133_Plus_2] Affymetrix Human Genome U133 Plus 2.0 Array platform. They were merged into a new dataset named GSEnew (*N* = 233), with batch effects eliminated, using the “sva” R package.

**TABLE 1:** TCGA pan-cancer description.

**Abbreviation**	**Number**	**Full name**
ACC	79	Adrenocortical carcinoma
BLCA	396	Bladder urothelial carcinoma
BRCA	1092	Breast invasive carcinoma
CESC	301	Cervical squamous cell carcinoma and endocervical adenocarcinoma
CHOL	36	Cholangiocarcinoma
COAD	280	Colon adenocarcinoma
DLBC	47	Lymphoid neoplasm diffuse large B-cell lymphoma
ESCA	183	Esophageal carcinoma
GBM	167	Glioblastoma multiforme
HNSC	516	Head and neck squamous cell carcinoma
KICH	65	Kidney chromophobe
KIRC	530	Kidney renal clear cell carcinoma
KIRP	270	Kidney renal papillary cell carcinoma
LAML	163	Acute myeloid leukemia
LGG	506	Brain lower-grade glioma
LIHC	361	Liver hepatocellular carcinoma
LUAD	497	Lung adenocarcinoma
LUSC	483	Lung squamous cell carcinoma
MESO	87	Mesothelioma
OV	264	Ovarian serous cystadenocarcinoma
PAAD	179	Pancreatic adenocarcinoma
PCPG	184	Pheochromocytoma and paraganglioma
PRAD	493	Prostate adenocarcinoma
READ	95	Rectum adenocarcinoma
SARC	249	Sarcoma
SKCM	463	Skin cutaneous melanoma
STAD	409	Stomach adenocarcinoma
TGCT	139	Testicular germ cell tumors
THCA	504	Thyroid carcinoma
THYM	118	Thymoma
UCEC	548	Uterine corpus endometrial carcinoma
UCS	57	Uterine carcinosarcoma
UVM	80	Uveal melanoma

### Identification of the Immune-Related eRNA-mRNA Axis

**FIGURE 1:**
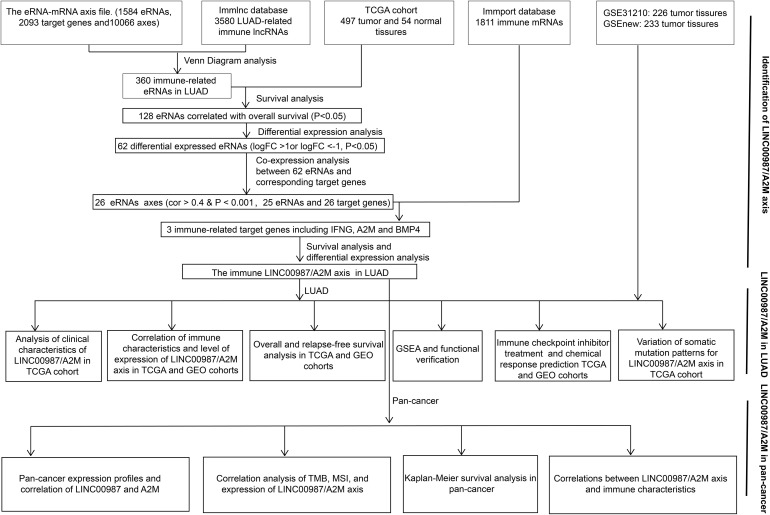
Workflow. Workflow for the identification and verification of the immune-related LINC00987/A2M axis in LUAD and pan-cancer.

The eRNA-mRNA axis file was obtained from the work of Vucicevic et al. (2015). The gene set of LUAD-related immune lncRNA was downloaded from ImmLnc^4^, whereas that of immune-related mRNA was obtained from the ImmPort database^5^. Using Venn diagram analysis, coexpression analysis (cor > 0.4 and *p* < 0.001), differential analysis (| log_2_FC| > 1 and *p* < 0.05), and survival analysis (*p* < 0.05), we finally identified only a single immune-related eRNA-mRNA axis associated with LUAD (Figure 1).

### Implementation of Single-Sample Gene Set Enrichment Analysis

Briefly, 29 immune-related gene sets containing immune cell types, immune-related pathways, and immune-related functions were derived from our previous study (Tang et al., 2020b). In addition, 26 stem cell gene sets were also obtained from our previous study (Tang et al., 2020a), whereas five mRNA-based hypoxia signatures were collected from the study of Winter et al. (2007), Buffa et al. (2010), Ragnum et al. (2015), Eustace et al. (2013), and Sorensen et al. (2010). Based on TCGA and GEO datasets, we applied the single-sample gene set enrichment analysis (ssGSEA) algorithm to quantify each signature’s enrichment scores using the “GSVA” package. The enrichment scores reflected the level of immune cell infiltration, degree of tumor hypoxia, and tumor cell stemness characteristics. Based on these enrichment scores, the relationships between the LINC00987-A2M axis and immunity, stem cell characteristics, and hypoxia were identified.

### Heatmap and Analysis of Significant Differences

The heatmaps of the ssGSEA score were visualized using the “heatmap” R package. The Wilcoxon test was used to calculate the significance of each signature score for eRNA and its target gene between the high- and low expression groups. Finally, we marked the significance of the heatmap values to show the difference between groups.

### Survival Analysis

Kaplan-Meier survival curves were constructed for relapse-free survival (RFS) and overall survival (OS) by using the “survminer” R package. Differences between groups were determined using the log-rank test. A *p*-value < 0.05 was considered statistically significant.

### Relationship Between LINC00987-A2M and Tumor Immunity

Based on the ESTIMATE algorithm (Yoshihara et al., 2013) and using gene expression profiles, we calculated the stromal score, immune score, ESTIMATE score, and tumor purity to verify the correlation between the proportion of immune cells and systemic content and LINC00987-A2M. We further evaluated the relationship between the infiltration degree of 22 immune cells and LINC00987-A2M based on analysis with the CIBERSORT algorithm (Newman et al., 2015), and only data with a CIBERSORT *p*-value < 0.05 were selected for consecutive analysis. Both the TMB and MSI have been associated with an increased response rate to immunotherapy (Narayanan et al., 2019; Liu J. et al., 2020), so we calculated the correlation between LINC0097-A2M and these indicators to evaluate the effectiveness of immunotherapy.

### Gene Set Enrichment Analysis

Hallmark gene sets are coherently expressed signatures that represent well-defined biological states or processes. We identified the biological effects caused by the change in the expression of LINC00987-A2M using h.all.v7.1.symbols.gmt [Hallmarks] (GSEA version 4.0.1). The analysis was performed using 1000 permutations, and a false discovery rate < 0.05 was set as the screening threshold.

### Prediction of Immunotherapeutic and Chemotherapeutic Response

Based on the subclass mapping method, we used the TCGA LUAD FPKM RNA-seq expression profile to predict the drug response of the LINC00987-A2M axis to immune checkpoint blockade. Predictive analysis of the chemotherapeutic response of LINC00987-A2M was performed based on the Genomics of Drug Sensitivity in Cancer (GDSC)^6^ using the “pRRophetic” package in R, where the half-maximum inhibitory concentration (IC50) of the sample was estimated using ridge regression, and the accuracy of the prediction was evaluated using 10-fold cross-validation, according to the GDSC training set. All parameters were set at default values, and the repeated gene expression was averaged. Three commonly used chemotherapeutic agents of LUAD were selected (Lu et al., 2019).

### Statistical Analyses

Wilcoxon test was used to assess changes in LINC00987 and A2M expression between cancer tissues and adjacent normal tissues and to compare the immune characteristics and estimated IC50 of the high- and low-expression groups for these two genes. Spearman’s and Pearson’s correlation tests were used for the analyses. All statistical analyses were performed using R 3.5.3, and *p* < 0.05 was considered statistically significant.

## Results

### Screening of Immune-Related eRNA-mRNA Axis Associated With LUAD

Using Venn diagram analysis, we obtained 360 immune **eRNA**s from the LUAD-related immune RNA and the eRNA-mRNA axis file, with 128 eRNAs associated with survival rate. The coexpression analysis results showed that out of 128 pairs of eRNA-mRNA axes, 53 pairs had a significant correlation between eRNAs and target genes. After differential analysis and survival analysis screening, we further obtained 16 differentially expressed and survival-related mRNAs. Besides, we found that when 16 mRNAs were again intersected with 1811 immune genes in the ImmPort database, only A2M was identified screened as an immune gene. Finally, we screened A2M and the upstream LINC00987 for subsequent immunological studies (Figure 1).

### Downregulation of LINC00987/A2M Axis Portended Adverse LUAD Pathological Progression and Worse Survival

**FIGURE 2:**
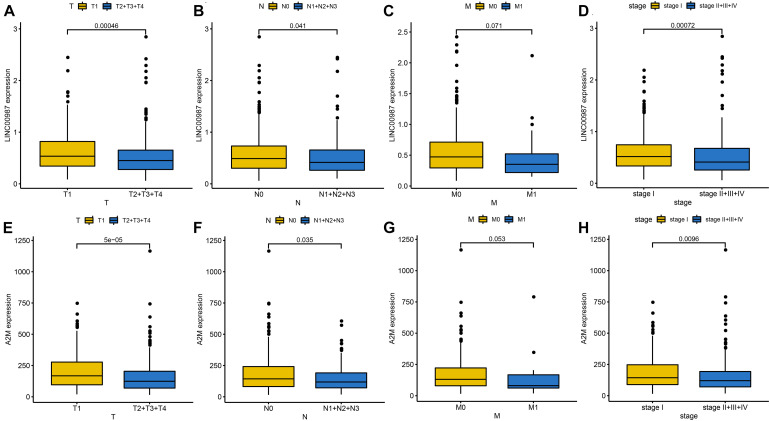
Identification of clinical characteristics of the two immune-related genes in the LUAD TCGA cohort. Differential expression of LINC00987 **(A–D)** and A2M **(E–H)** among the T (tumor), N (node), M (metastasis), and stage, evaluated using the Wilcoxon test.

We employed the “ggpubr” package to investigate the impact of the LINC00987/A2M axis on clinical characteristics in the TCGA cohort. We accordingly observed that the expression levels of LINC00987 and A2M were both inversely correlated with T (tumor), N (node), and stage; however, M (metastasis) was not affected by either (Figures 2A–H).

**FIGURE 3:**
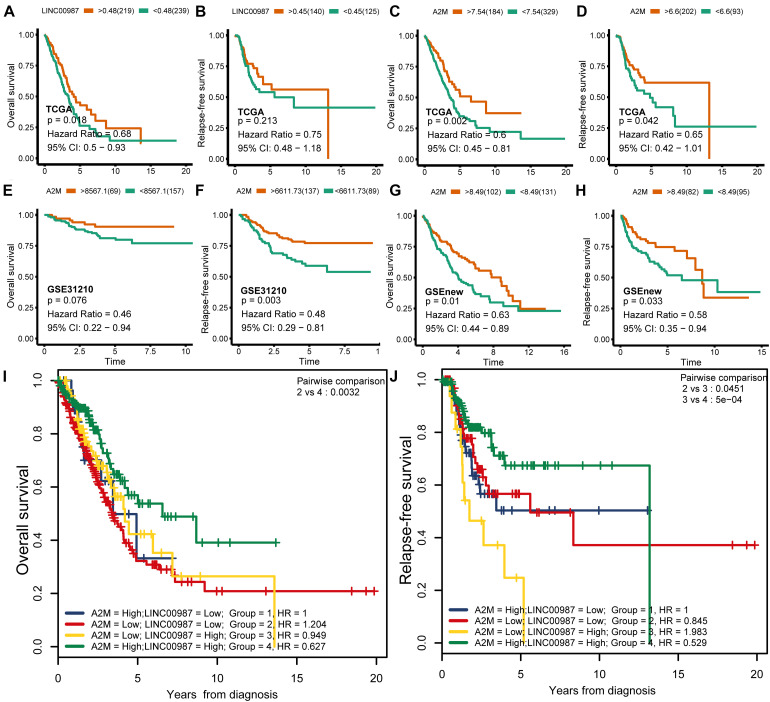
Correlation of overall (OS) and relapse-free survival (RFS) with LINC00987 and A2M in datasets. **(A–D)** OS and RFS curves for LINC00987 and A2M in the TCGA cohort. **(E–H)** OS and RFS curves for A2M in the GSEnew and GSE31210 cohorts. **(I,J)** OS and RFS curves for combinations of LINC00987 and A2M in the TCGA cohort.

We speculated that patients with low expression of LINC00987 or A2M were more likely to have malignant pathology than patients with high expression. Kaplan–Meier analysis also showed that LINC00987 and A2M were protective predictors of OS (LINC00987: HR = 0.68, 95% CI [0.5–0.93], and *p* = 0.018; A2M: HR = 0.6, 95% CI [0.45–0.81], and *p* = 0.002) and RFS (LINC00987: HR = 0.62, 95% CI [0.39–0.99], and *p* = 0.037; A2M: HR = 0.59, 95% CI [0.36–0.98], and *p* = 0.025; Figures 3A–D). To further identify their impact on the survival prognostic values in patients with LUAD, we downloaded and integrated 3 GEO datasets (GSE31210, GSE37745, and GSE50081). We found that patients with high expression of A2M had longer OS (GSE31210: HR = 0.38, 95% CI [0.18–0.78], and *p* = 0.037; GSEnew: HR = 0.63, 95% CI [0.44–0.89], and *p* = 0.01) and RFS (GSE31210: HR = 0.48, 95% CI [0.29–0.81], and *p* = 0.003; GSEnew: HR = 0.58, 95% CI [0.35–0.94], and *p* = 0.033) than patients in the low expression group (Figures 3E–H). Combination group analysis revealed that patients with high expression of both genes had better OS (*p* < 0.05) and RFS (*p* < 0.05) among all four groups (Figures 3I,J).

### Correlation of Immune Characteristics and Level of Expression of LINC00987/A2M Axis

**FIGURE 4:**
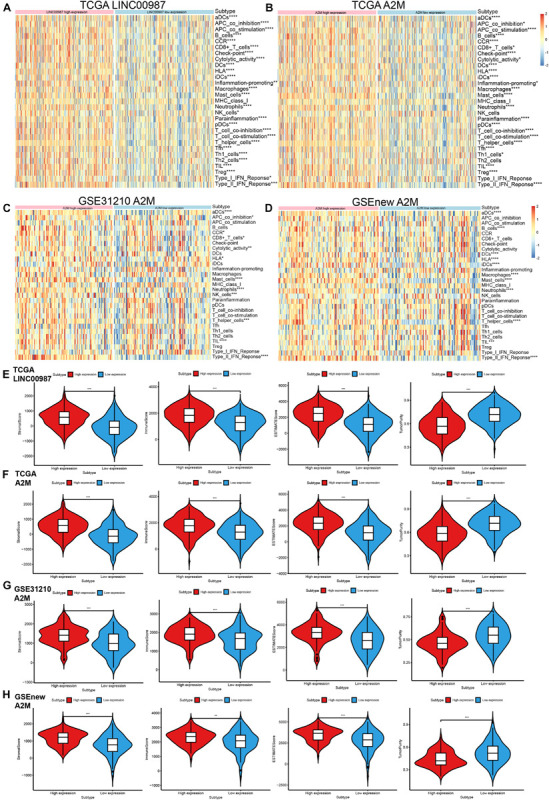
Distribution of immune-related characteristics in different subgroups related to LINC00987 and A2M in the TCGA and GEO datasets. **(A,B)** Heatmap of the expression of LINC00987 and A2M and 29 immune-related gene sets generated by ssGSEA in TCGA cohort. **(C,D)** Heatmap of the expression of A2M and 29 immune-related gene sets generated by ssGSEA in two GEO cohorts. Violin-plot showing the difference in ImmuneScore, StromalScore, ESTIMATEScore, and TumorPurity in **(E)** LINC00987 and **(F–H)** A2M subgroups in TCGA and GEO cohorts. *p < 0.05, **p < 0.01, ***p < 0.001, and ****p < 0.0001.

The LINC00987/A2M axis was shown to be involved in LUAD immunity, but its relation to specific processes and the extent of participation remained unknown. We, therefore, focused on deciphering these aspects. We used the ssGSEA scores of 29 immune gene sets to evaluate the relationship between immune infiltration and LINC00987 or A2M expression in the low- and high- LINC00987 or A2M expression groups (Figures 4A–D).

Among these 3 cohorts, we found that most immune cell infiltration degree was significantly different in the TCGA cohort but only partially significant in the GEO datasets. This might have resulted from the small sample size and large error in the GEO datasets. Our ESTIMATE analysis demonstrated a high proportion of immune cells and systemic response in high LINC00987 and A2M expression groups among the three cohorts, reflected by higher stromal score, immune score, ESTIMATE score, and lower tumor purity (Figures 4E–H).

**FIGURE 5:**
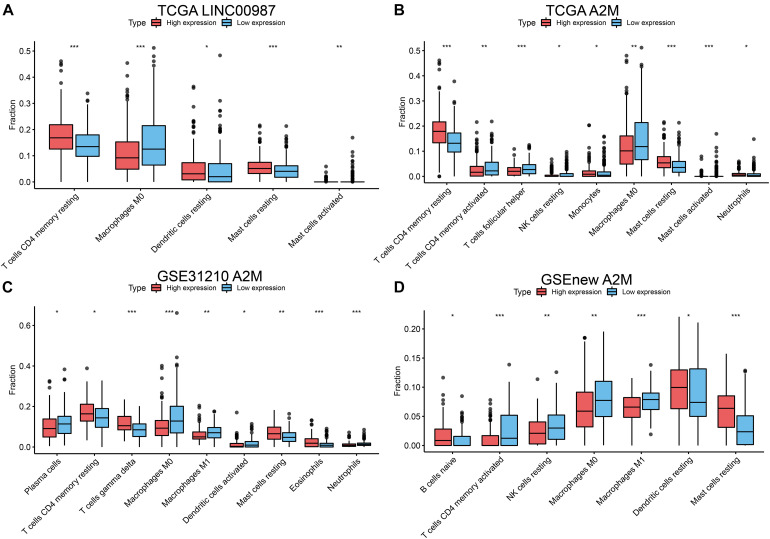
Differences in the distribution of 22 human immune cells in LINC00987 and A2M expression groups estimated using the CIBERSORT algorithm. Boxplots for **(A)** LINC00987 and **(B)** A2M in the TCGA cohort. Boxplots for A2M in the **(C)** GSE31210 and **(D)** GSEnew cohorts. *p < 0.05, **p < 0.01, and ***p < 0.001.

We further evaluated the relationship between the differential distribution of 22 human immune cell subgroups and the LINC00987/A2M axis based on the CIBERSORT algorithm. We observed that the populations of 22 immune cells in different active states, including CD4 memory T cells, M0 macrophages, and mast cells, showed significant differences in both the high LINC00987 and A2M expression groups compared with the low expression groups (Figures 5A–D). This finding suggested that the LINC00987/A2M axis might mainly regulate immune cell infiltration. In general, the above results consistently indicated that when the LINC00987/A2M axis is highly expressed, immune cells in the tumor microenvironment tend to be more abundant and characterized by increased immune cell components, which might contribute to the better prognosis of patients.

### GSEA and Functional Verification

**FIGURE 6:**
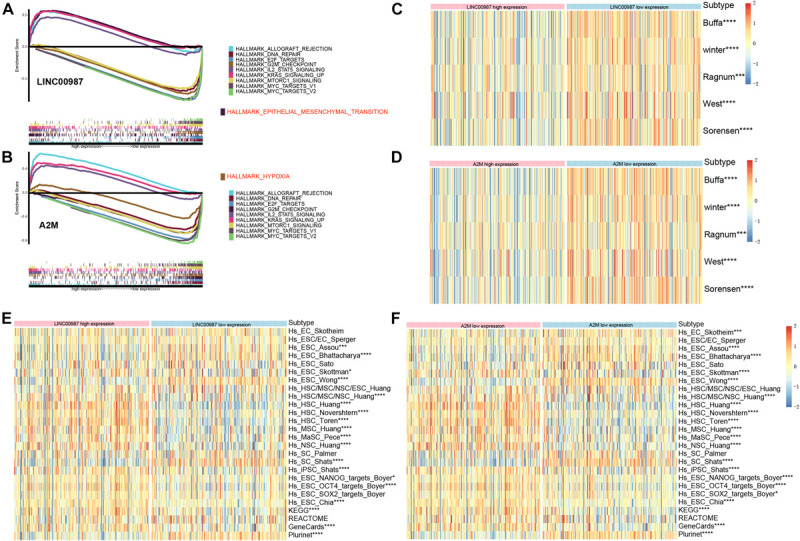
Gene sets functional enrichment analysis. **(A)** GSEA of high vs. low expression of LINC00987. **(B)** GSEA of high vs. low expression of A2M in the TCGA cohort. Correlation of the expression of **(C)** LINC00987 and **(D)** A2M with hypoxia gene sets. Correlation of the expression of **(E)** LINC00987 and **(F)** A2M with tumor stem cell characteristics. *p < 0.05, ***p < 0.001, and ****p < 0.0001.

We performed Gene set enrichment analysis (GSEA) to assess the biological effects of the changes in LINC00987/A2M expression in LUAD. The hallmarks of the expression of LINC00987 and A2M are illustrated in Figures 6A,B. We observed that these hallmarks, including allograft rejection, DNA repair, E2F targets, G2M checkpoint, IL2-STAT5 signaling, Kras signaling, Mtorc1 signaling, and MYC targets, were consistent for the expression of both LINC00987 and A2M. However, LINC00987 and A2M were also shown to be related to epithelial-mesenchymal transition and hypoxia, respectively (Figures 6A,B). To further explore whether the immune-related LINC00987/A2M axis also affected tumor epithelial-mesenchymal transition and hypoxia, we analyzed the relationship between the five hypoxia-associated gene sets and the LINC00987/A2M axis in TCGA, GSE31210, and GSEnew cohorts. The ssGSEA scores of all five hypoxia-associated gene sets indicated that they were up-regulated in the low LINC00987 or A2M expression group, implying that the LINC00987/A2M axis facilitated tumor cell growth and proliferation under hypoxia (Figures 6C,D and Supplementary Figures 1A,B). Based on the 26 stem cell gene sets, we found that the distribution of the ssGSEA score related to various stemness characteristics was significantly different between high and low LINC00987 or A2M expression groups (Figures 6E,F and Supplementary Figures 1C,D).

### Differences in Sensitivity of the LINC00987/A2M Axis to Checkpoint Inhibitors and Chemotherapy

**FIGURE 7:**
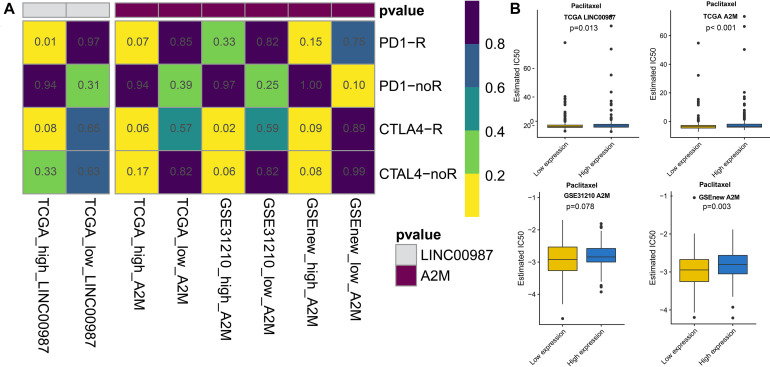
Response to immunotherapy and sensitivity to chemotherapy in relation to the expression of LINC00987 and A2M in TCGA, GSE31210, and GSEnew cohorts. **(A)** Sensitivity response of high vs. low expression of LINC00987 to PD1 and CTLA-4 inhibitors (*p* = 0.01). **(B)** Boxplots of the estimated IC50 for paclitaxel in high vs. low expression of LINC00987/A2M.

With the approval of immune checkpoint inhibitors in cancer therapy, it is vital to understand the immune checkpoint regulation mechanism to improve their efficacy. We observed that the high LINC00987 and A2M expression groups were more sensitive to anti-PD-1 treatment in TCGA and GSE31210 cohorts (*p* < 0.05; Figure 7A), suggesting that the LINC00987/A2M axis is an effective sensitizer for the curative effect. In addition, compared to the conventional chemotherapy of LUAD, we found that variations in the LINC00987/A2M axis heralded different responses to immunotherapy. We evaluated the sensitivity of commonly used chemotherapeutic drugs in treating LUAD in each sample in TCGA and GEO cohorts by estimating the IC50. We accordingly observed that the low LINC00987 and A2M expression groups were more sensitive to treatment with paclitaxel than the high expression groups in TCGA and GSEnew cohorts (*p* < 0.05; Figure 7B).

### Variation of Somatic Mutation Patterns for LINC00987/A2M Axis in LUAD

**FIGURE 8:**
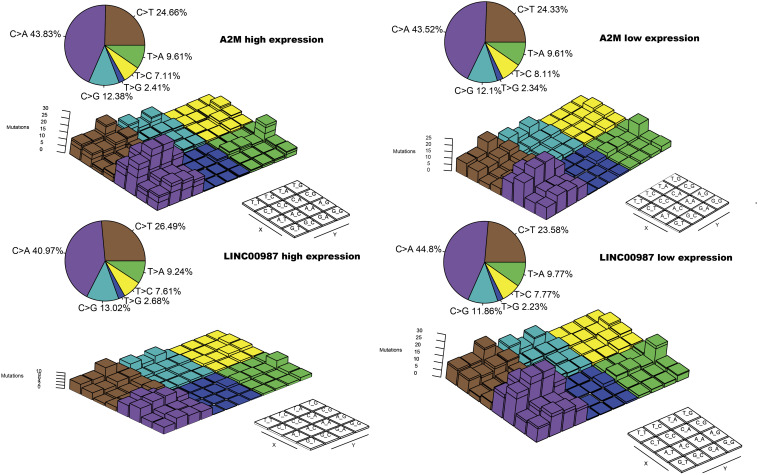
Lego plot representation of mutation patterns in LUAD samples in relation to the LINC00987/A2M axis. Single-nucleotide substitutions are divided into six categories with 16 surrounding flanking bases. The pie chart in the upper left shows the proportion of six categories of mutation patterns.

To gain further insights into the mutational processes in high and low expression of LINC00987 or A2M, we delineated the mutation patterns from the data on somatic mutations in LUAD. We noted that the overall mutational pattern was mainly dominated by C > T and C > A mutations. In particular, C > A mutations were shown to be increased, whereas C > T, and C > G mutations were decreased in the high LINC00987 expression group. However, these alterations were not observed in the high A2M expression group (Figure 8 and Supplementary Figure 2).

### Pan-Cancer Expression Profiles and Correlation of LINC00987 and A2M

**FIGURE 9:**
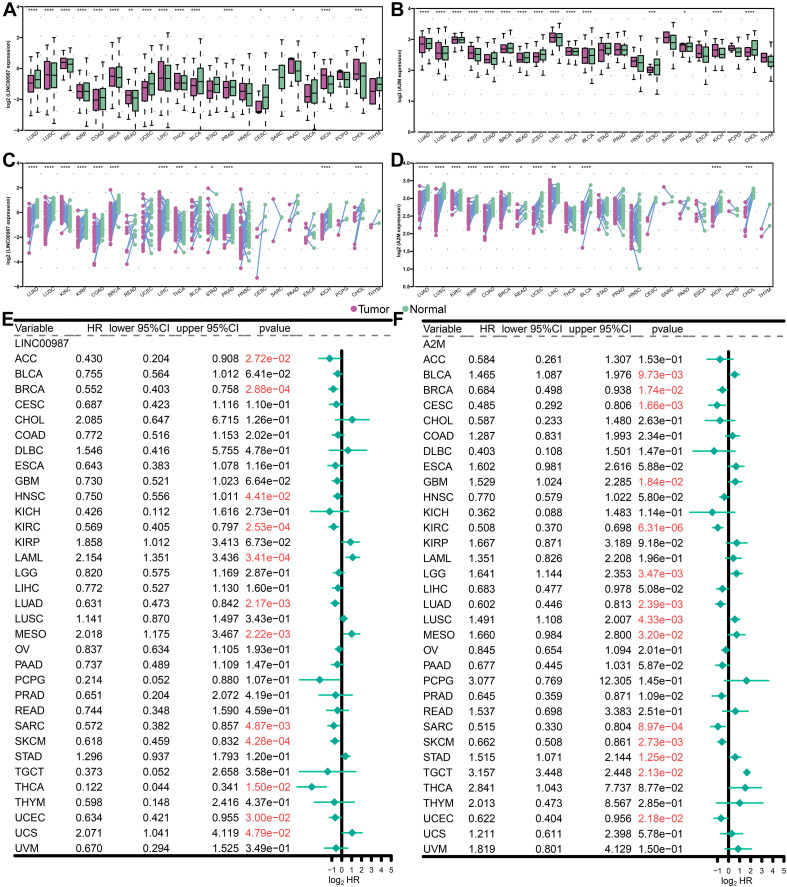
Levels of expression and Kaplan-Meier survival analysis of LINC00987 and A2M in pan-cancer analysis. **(A,B)** Population differences in the expression of LINC00987 and A2M. **(C,D)** Pairing differences in the expression of LINC00987 and A2M. **(E,F)** Red values indicate statistical significance of the *p* value (*p* < 0.05). HR hazard ratio, 95% CI 95% confidence interval, *p < 0.05, **p < 0.01, ***p < 0.001, and ****p < 0.0001.

Multiple lines of evidence are required to confirm the role of the LINC00987/A2M axis in tumor immunity. Therefore, we used the UCSC Xena datasets to investigate the pan-cancer expression profiles of LINC00987 and A2M and their prognostic significance. Cancer tissues lacking paired normal tissues were not included. We accordingly found that the levels of LINC00987 and A2M were consistently down-regulated in most tumor tissues in comparison with non-carcinoma tissues, both in overall and paired comparison of 22 cancer types, including LUAD, lung squamous cell carcinoma (LUSC), kidney renal papillary cell carcinoma (KIRP), colon adenocarcinoma (COAD), breast invasive carcinoma (BRCA), uterine corpus endometrial carcinoma (UCEC), urothelial bladder carcinoma (BLCA), cholangiocarcinoma (CHOL), and kidney chromophobe. However, we noted that both were up-regulated in prostate adenocarcinoma (PAAD). These two genes were shown not to be differentially expressed or unrelated in individual cancers (Figures 9A–D).

We further analyzed the association between the expression levels of LINC00987 and A2M and survival by using Kaplan–Meier survival analysis. We demonstrated that LINC00987 and A2M could be consistently regarded as protective factors in BRCA, kidney renal clear cell carcinoma, LUAD, sarcoma (SARC), cutaneous skin melanoma (SKCM), and UCEC; however, both were revealed to be risk factors for mesothelioma (MESO; Figures 9E,F). Combined with the above verification results, we assumed that the LINC00987/A2M axis might play key roles in cancer progression for LUAD, BRCA, and UCEC. In addition, the expression of LINC00987 and A2M was demonstrated to have an obvious correlation in almost all tumors (Supplementary Table 1).

### Correlation Analysis of TMB, Microsatellite Instability, and Expression of LINC00987/A2M Axis

**FIGURE 10:**
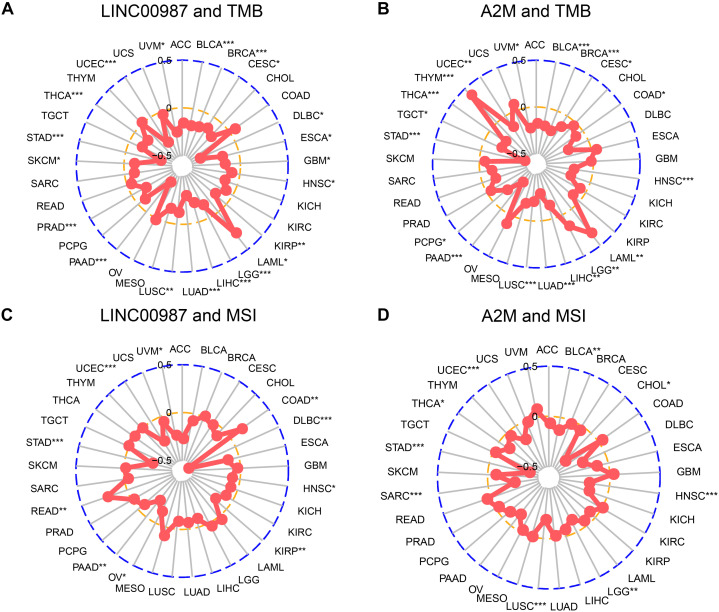
Correlation of alterations in TMB and MSI with the level of expression of LINC00987 and A2M in various tumors. **(A,C)** Radar chart showing the correlation between LINC00987 and TMB **(A)** and MSI **(C)** in 33 cancer types. **(B,D)** Relationship between A2M and TMB **(B)** and MSI **(D)**. *p < 0.05, **p < 0.01, and ***p < 0.001.

We next examined the associations between the expression of LINC00987/A2M and the levels of TMB and MSI (Figure 10). We found that TMB showed differences related to the expression of LINC00987 in BRCA, BLCA, cervical squamous cell carcinoma and endocervical adenocarcinoma (CESC), UCEC, uveal melanoma (UVM), thyroid carcinoma (THCA), stomach adenocarcinoma (STAD), SKCM, prostate adenocarcinoma (PRAD), pancreatic adenocarcinoma (PAAD), LUSC, LUAD, liver hepatocellular carcinoma (LIHC), brain lower-grade glioma (LGG), acute myeloid leukemia (LAML), KIRP, HNSC, esophageal carcinoma (ESCA), and lymphoid neoplasm diffuse large B-cell lymphoma (DLBC; Figure 10A). The expression of A2M was shown to be correlated with TMB in BRCA, BLCA, COAD, CESC, HNSC, UCEC, UVM, THCA, thymoma (THYM), TGCT, STAD, pheochromocytoma, and paraganglioma, PAAD, LUSC, LUAD, LIHC, LGG, and LAML (Figure 10B). The expression of LINC00987 was found to be correlated with MSI in UVM, COAD, DLBC, HNSC, KIRP, ovarian serous cystadenocarcinoma (OV), PAAD, rectum adenocarcinoma, STAD, and UCEC (Figure 10C), whereas the expression of A2M was correlated with MSI in BLCA, CHOL, HNSC, LGG, LUSC, SARC, STAD, THCA, and UCEC (Figure 10D).

### Correlations Between LINC00987/A2M Axis and Immune Cell Infiltration via Pan-Cancer Analysis

**FIGURE 11:**
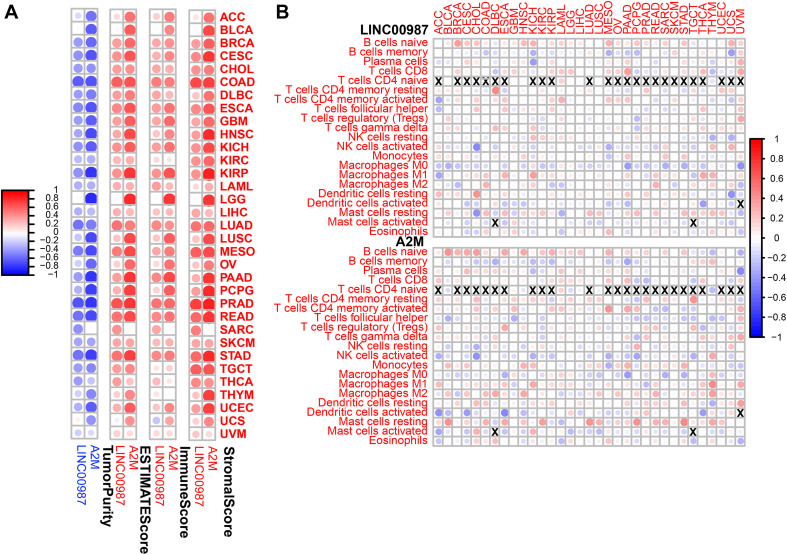
Immune regulation according to the expression of LINC00987 and A2M in the pan-cancer analysis. **(A)** Correlation between the level of expression of LINC00987 and A2M and the immune score in multiple tumors determined by ESTIMATE. **(B)** Correlation between the expression of LINC00987/A2M and infiltration by 22 types of immune cells in pan-cancer analysis. Red indicates a correlation coefficient > 0, whereas blue indicates a correlation coefficient < 0. *p* < 0.05.

Subsequently, we conducted a pan-cancer analysis to examine the association between the proportion of immune cells and their systemic content with LINC00987/A2M expression. We observed that nearly all of the tumors studied had a high degree of immune infiltration with a high stromal score, immune score, ESTIMATE score, and low tumor purity (Figure 11A). Furthermore, most immune cell distribution and activity in the immune microenvironment showed a similar trend (Figure 11B). Similar to the results analyzed in LUAD, resting mast cells and CD4 resting memory T cells were demonstrated to be up-regulated in most tumors, whereas the activation of memory M0 macrophages was inhibited.

## Discussion

Our results revealed the abnormal expression and mutual regulation of the LINC00987/A2M axis. We showed a correlation between the LINC00987/A2M axis expression and disease progression in LUAD. More specifically, patients with low LINC00987/A2M axis expression showed more aggressive malignancy and faster progression, as well as reduced OS and shorter RFS. Wang et al. reported that LINC00987 expression was down-regulated in COPD tissues and LPS-induced BEAS-2B cells. LINC00987 was transfected into LPS-induced 16HBE and BEAS-2B cells with a control group. LINC00987 protected 16HBE and BEAS-2B cells from LPS-induced apoptosis, oxidative stress, inflammation, and autophagy (Wang et al., 2020). In our research, LINC00987 upregulation was found to be deeply involved in immune system events. In terms of cellular components, LINC00987 upregulation involves the composition of immune receptors (IMMUNOLOGICAL_SYNAPSE, and T_CELL_RECEPTOR_COMPLEX, etc.); while regarding biological functions, LINC00987 upregulation is involved with a combination of chemokines, cytokines, immune receptors, and immunoglobulins. In terms of biological function, LINC00987 upregulation positively correlated with the proliferation and differentiation of B and T cells, as well as T cell activation. Therefore, in general, the upregulation of LINC00987 not only involves the recruitment of immune cells, antigen recognition, and presentation but also participates in the effect of immune cells on target cells (antitumor effects). Simultaneously, LINC00987 downregulation significantly affects mitochondrial translation, cell respiration, and other energy metabolism processes. The functional enrichment analysis of A2M was highly similar to that of LINC00987, as shown in Supplementary Figure 3. The A2M protein is known as an acute-phase protein of the innate immune system, acting as a protector in withstanding stress and inflammation in early age (Birkenmeier et al., 2003; Mocchegiani et al., 2007; Xue et al., 2017). α2-macroglobulin (α2M) and related proteins share the function of binding to the host or foreign peptides and particles, thereby becoming a humoral defense barrier against pathogens in vertebrate plasma and tissues (Borth, 1992). At present, the beneficial impact of a transformation-associated isoform of A2M on tumors and other diseases has been mainly discussed in terms of clearing growth factors, especially TGF-β1, which strongly promotes the malignancy of glioma (Lauer et al., 2001; Wick et al., 2001; Sinnreich et al., 2004). Lindner et al. (2010) discovered that the isoform of α2-macroglobulin A2M and its interaction with low-density lipoprotein receptor-related protein 1 (LRP1) inhibit tumor cell proliferation through the Wnt/β-catenin signaling pathway, migration, invasion, spheroid formation, and anchoring mechanisms for uncontrolled growth. Nevertheless, Alpha-2 macroglobulin (A2M) acts as a general protease inhibitor in serum and can bind various cytokines and growth factors. Taking advantage of the immunoaffinity of the A2M protein complex in human serum, more and more studies are using A2M protein complex as a new serum biomarker for cancer (Zhang et al., 2000; Kanoh et al., 2001; Burgess et al., 2008).

**FIGURE 12:**
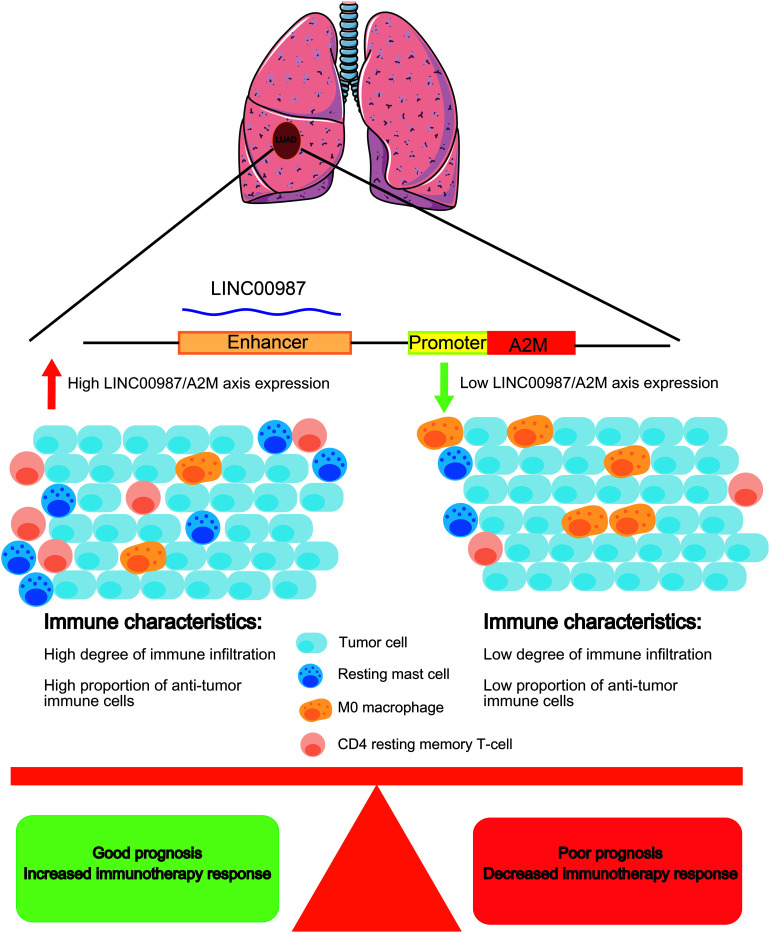
Schematic diagram. LUAD patients with a high LINC00987/A2M axis expression have a better prognosis, with an increased proportion of antitumor immune cell infiltration. Immunotherapy may improve the prognosis of LUAD patients with a high LINC00987/A2M axis expression.

The high expression of LINC00987 and A2M was associated with the infiltration of numerous immune cells, high immune score signatures, and low tumor purity in multiple analyses of immune cell infiltration and scoring. For instance, CD4 resting memory T-cells and resting mast cells were shown to be increased in the high LINC00987 and A2M expression groups, whereas M0 macrophages were decreased. We found that both LINC00987 and A2M significantly affected the distribution of immune cells in the immune microenvironment of patients with LUAD. Mast cells play a key role in the infiltration of the tumor microenvironment by immune cells, during which they have been reported to release proangiogenic factors to promote angiogenesis and tumor development (Sammarco et al., 2019). Tumors lacking central memory CD4 T-cells were associated with better prognosis, consistent with our research (Wu et al., 2019). Tumor-associated macrophages (TAMs) are categorized into two subtypes: M1 macrophages that are known to exhibit an antitumor effect, and M2 macrophages that have been reported to play a role in tumor promotion. Moreover, M1 and M2 macrophages can transform each other with the appropriate stimuli (Kim and Bae, 2018; Zhihua et al., 2019). Our results showed that the number of M0 macrophages decreased when LINC00987 and A2M were highly expressed, which might have been the result of the transformation of M0 to M1 or M2 cells. According to our results, LINC00987 and A2M might inhibit tumor growth by suppressing mast cells and memory CD4 T-cells and promoting the conversion of M0 macrophages to M1 and M2 (Figure 12). However, the specific mechanism requires further study.

In GSEA, both LINC00987 and A2M were demonstrated to be enriched in immunoreaction, immune checkpoint, and STAT5/Kras cancer signaling pathways. This finding suggested that putatively eRNA-regulated target genes consisting of clinically actionable genes and immune checkpoints can also affect immunotherapy and drug response (Chen et al., 2016). We also found that patients with high LINC00987 and A2M expression had increased sensitivity to treatment with inhibitors of the PD1 and CTLA-4 immune checkpoints. This discovery characterized the therapeutic potential of the eRNA-mRNA axis against cancer in the clinical setting. Our GSEA analysis showed that LINC00987 might regulate the epithelial-mesenchymal transition process of tumors; however, due to the lack of relevant EMT gene sets for verification and the close relationship between epithelial-mesenchymal transition and tumor stemness (Mani et al., 2008), we used stem gene sets for verification. We thus found that the LINC00987/A2M axis might play an important role in regulating tumor stemness and tumor hypoxia. Besides, we noted that the degree of tumor hypoxia and stemness characteristics were greatly influenced by LINC00987 and A2M. In particular, tumor cells in a hypoxic state were shown to continue to differentiate, with increases in neovascularization and abnormal activation of most TF, promoting cancer occurrence and metastasis. Moreover, it has been shown that hypoxic areas of solid tumors were infiltrated by a large number of immunosuppressive cells during hypoxia, influencing the antitumor immune response by promoting local immunosuppression (Noman et al., 2011). Therefore, we hypothesized that although LINC00987 and A2M are immune-related genes, they potentially promote an immune cell infiltration and antitumor immune response by suppressing the degree of hypoxia. Tumor cells have been reported to transmit inhibitory signals through immune checkpoints, inhibit the function of T cells, induce the apoptosis of T cells, and cause immune escape. However, the overexpression of LINC00987 and A2M might have caused resistance to paclitaxel. Our GSEA results showed that both LINC00987 and A2M had a regulatory effect on the G2M checkpoint. Paclitaxel-induced tumor cell death occurs without a prior G2/M-phase arrest, and thus LINC00987/A2M reduces the sensitivity to paclitaxel treatment by regulating the cell cycle (Dziadyk et al., 2004). This finding might help determine patients’ response with LUAD to immunotherapy and their sensitivity to chemotherapy drugs (Figure 12).

Mutations can be the beginning of tumorigenesis, but they may also initiate self-destruction (Weinberg, 2011). We have known that tumors with increased numbers of somatic mutations, known as TMB, are characterized by the production of more neoantigens than could be recognized by T cells and have been reported to be sensitive to PD-1/PD-L1 inhibitors (Rooney et al., 2015; Yarchoan et al., 2017). The neoantigen load positively correlated to cytolytic activity among multiple tumor types, with the cytolytic immune response being beneficial to effective natural antitumor immunity (Rooney et al., 2015). Previous research has identified that in KRAS-mutant LUAD, STK11/LKB1 mutations were major drivers of resistance to PD-1 blockade (Koulidis et al., 2018). In this study, we detected somatic mutations, in which the distribution of the C > A modality was increased in the high LINC00987 expression group, and TMB was highly correlated with the LINC00987/A2M axis. It could be speculated that the generation of somatic mutations when the LINC00987/A2M axis was highly expressed was conducive to increasing the production of neoantigens and promoting the therapeutic sensitivity of immune checkpoint inhibitors, thus constituting a breakthrough in the treatment of LUAD. Microsatellites are regions of short tandem repeats of 10 to 60 base pairs, including both coding and non-coding areas (Ross et al., 2003), regularly maintained by the mismatch repair (MMR) system in normal cells (Bonneville et al., 2017). Owing to disorders in the MMR system, replication errors such as insertion or loss of base pairs might occur, resulting in MSI, which is the inability to guarantee the stable length of the microsatellite (Bonneville et al., 2017). Microsatellites influence the expression of genes and directly or indirectly regulate the genome by being located and linked to several important gene sites that are markers of human disease and etiology. Therefore, MSI has become an important feature in many diseases, especially in tumors. This has been reflected in the discovery that patients with colorectal cancer with high MSI had a better prognosis than patients with microsatellite stable (MSS) and low MSI (Benatti et al., 2005; Buckowitz et al., 2005). In addition, mutations in the CASP8 and MHC Class I cytolysis effector molecules were the most enriched mutations in MSI-high tumors, highlighting MSI-high as a contributor to promoting antigen presentation and extrinsic apoptosis and in promoting natural antitumor immunity (Rooney et al., 2015). Although LINC00987 and A2 M expression did not correlate with MSI in LUAD, other cancers have been reported to exhibit varying MSI degrees. As such, LINC00987 and A2M might be key factors in treatment strategies against these cancers. The pan-cancer validation results showed that the LINC00987/A2M axis was widely down-regulated in most cancer tissues compared with that in corresponding normal tissues, and this abnormally low expression might be a risk factor for the worse OS of patients with these cancer types, except for MESO and PAAD. In our correlation analysis, the expression of the LINC00987/A2M axis was related to immune cell infiltration in 33 tumors, with A2M, in particular, exhibiting a higher and more obvious correlation. Among the 22 immune cell types, resting mast cells and CD4 resting memory T cells were positively correlated, whereas M0 macrophages negatively correlated with the expression of LINC00987/A2M. These results were consistent with LUAD and confirmed the LINC00987/A2M axis’s key role in immune regulation.

## Conclusion

In conclusion, the LINC00987/A2M axis was down-regulated in LUAD, indicating poor survival and malignant progression. The heterogeneity in immune cell infiltration, sensitivity to checkpoint inhibitors and chemotherapy, degree of tumor hypoxia, stemness characteristics, and somatic mutations were demonstrated to differ after abnormal expression of the LINC00987/A2M axis. Validation of different methods of estimation of the immune status in four datasets indicated that the LINC00987/A2M axis could offer valuable new biomarkers for the prognosis and immunotherapy of LUAD and most human cancers.

## Data Availability Statement

The original contributions presented in the study are included in the article/Supplementary Material, further inquiries can be directed to the corresponding author/s.

## Author Contributions

JM: conceptualization, methodology, software, visualization, writing, review, and editing. XL: conceptualization, writing, review, and editing. XW: conceptualization and supervision. QM: review and editing. TW: software and visualization. CT: conceptualization, methodology, and supervision. All authors contributed to the article and approved the submitted version.

## Conflict of Interest

The authors declare that the research was conducted in the absence of any commercial or financial relationships that could be construed as a potential conflict of interest.

## Footnotes

1 https://gdc-portal.nci.nih.gov/

2 http://xena.ucsc.edu/

3 https://www.ncbi.nlm.nih.gov/geo/

4 http://bio-bigdata.hrbmu.edu.cn/ImmLnc/

5 https://www.immport.org/

6 https://www.cancerrxgene.org/

## Supplementary Material

Supplementary Figure 1 Analysis of the functional characteristics of LINC00987/A2M. Correlation of the expression of **(A)** LINC00987 and **(B)** A2M with hypoxia gene sets. Correlation of the expression of **(C)** LINC00987 and **(D)** A2M with tumor stem cell characteristics. **p* < 0.05, ****p* < 0.01, ****p* < 0.001, and *****p* < 0.0001.

Supplementary Figure 2 Landscape of somatic mutation of LINC00987/A2M axis in TCGA. Top 20 genes in LINC00987 **(A)** high and **(B)** low expression group, A2M **(C)** high and **(D)** low expression group, respectively.

Supplementary Figure 3 Function enrichment analysis of LINC00987/A2M axis in GSEA. Kyoto Encyclopedia of Genes and Genomes (KEGG) for high and low expression groups of **(A)** LINC00987 and **(B)** A2M; Gene Ontology (GO) term of Biological Process (BP) for high and low expression groups of **(C)** LINC00987 and **(D)** A2M; cellular component (CC) for high and low expression groups of **(E)** LINC00987 and **(F)** A2M; molecular function (MF) for high and low expression groups of **(G)** LINC00987 and **(H)** A2M.
