# Supplementary figures and images for: Reconstruction and Analysis of the Immune-Related LINC00987/A2M Axis in Lung Adenocarcinoma
Source: Front Mol Biosci. 2021 Apr 27;8:644557. doi: 10.3389/fmolb.2021.644557 (PMC8111304; doi:10.3389/fmolb.2021.644557)

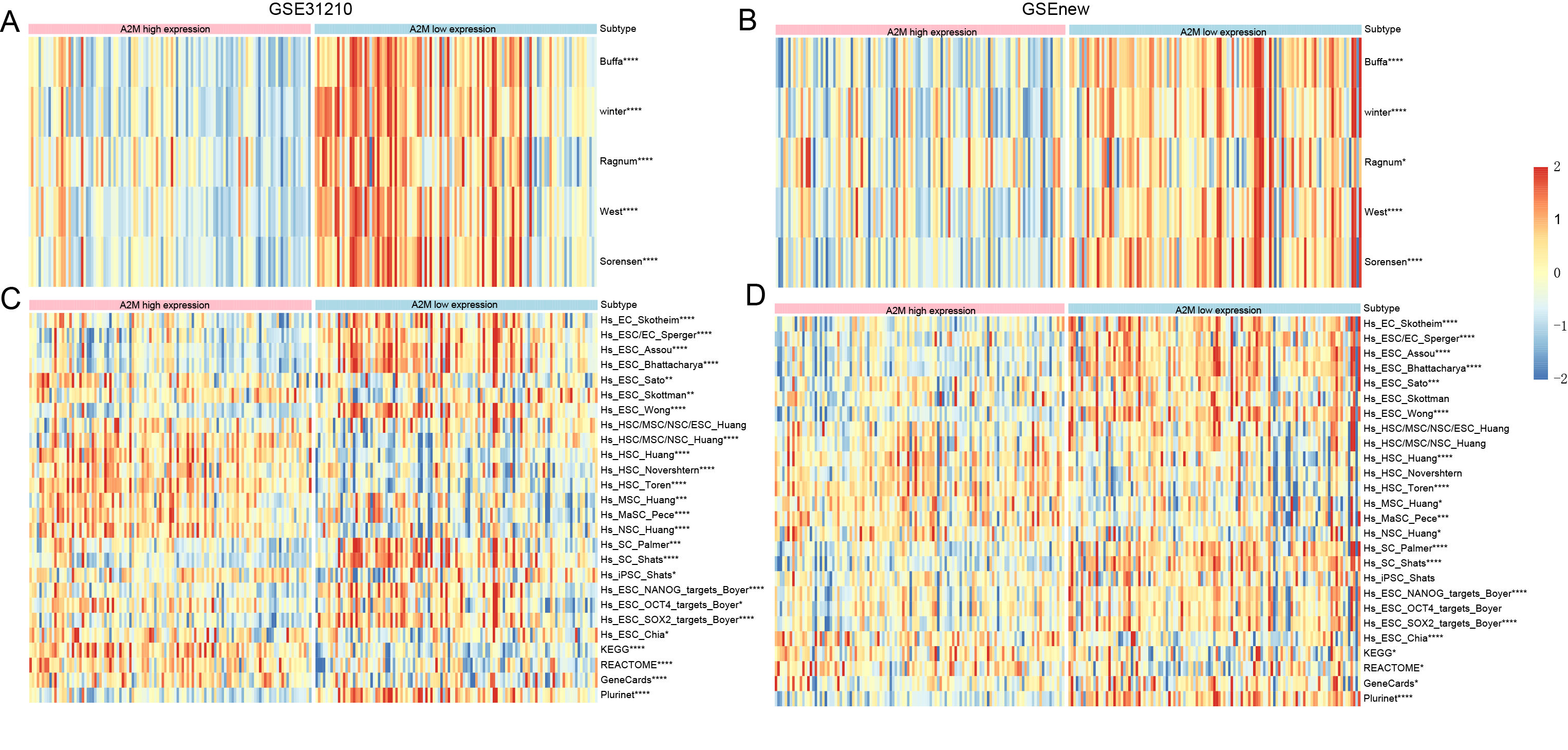

Supplement: Supplementary Figure 1 — Analysis of the functional characteristics of LINC00987/A2M. Correlation of the expression of (A) LINC00987 and (B) A2M with hypoxia gene sets. Correlation of the expression of (C) LINC00987 and (D) A2M with tumor stem cell characteristics. *p < 0.05, ***p < 0.01, ***p < 0.001, and ****p < 0.0001. [file Image_1.TIF]

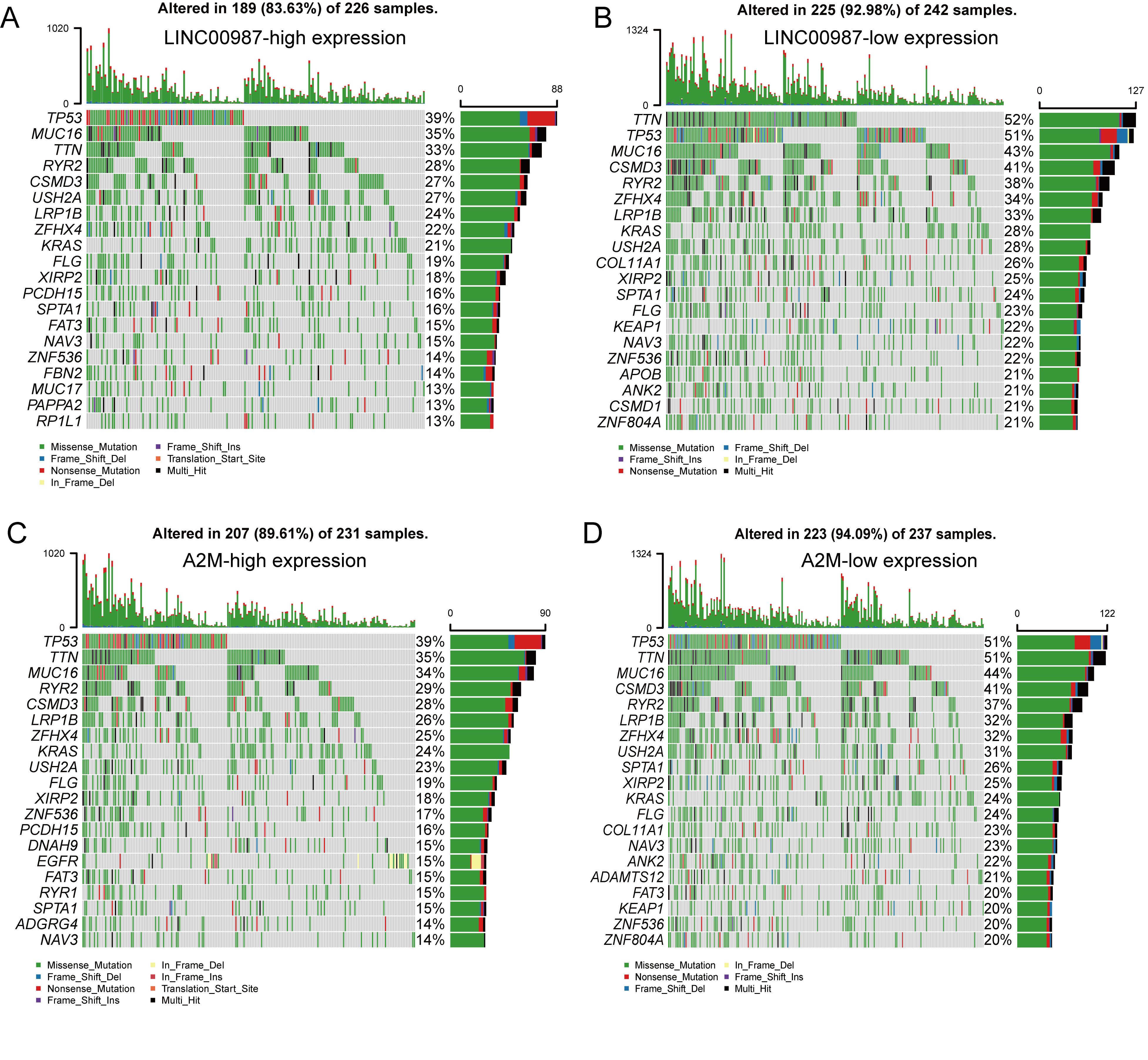

Supplement: Supplementary Figure 2 — Landscape of somatic mutation of LINC00987/A2M axis in TCGA. Top 20 genes in LINC00987 (A) high and (B) low expression group, A2M (C) high and (D) low expression group, respectively. [file Image_2.TIF]
